# Study of the Influence of Selected Carrageenan Fractions on the Physical Properties and Crystal Structure of Mango Sorbet

**DOI:** 10.3390/gels11070531

**Published:** 2025-07-09

**Authors:** Anna Kamińska-Dwórznicka, Ewa Gondek, Ewa Jakubczyk

**Affiliations:** Department of Food Engineering and Process Management, Institute of Food Sciences, Warsaw University of Life Sciences (WULS-SGGW), Nowoursynowska 159C, 02-776 Warsaw, Poland; ewa_gondek@sggw.edu.pl (E.G.); ewa_jakubczyk@sggw.edu.pl (E.J.)

**Keywords:** sorbet, carrageenan fractions, stabilizers, crystal structure

## Abstract

The aim of this study was to evaluate the effect of the iota, kappa and lambda carrageenan fractions on the physical properties and crystal structure of a fruit sorbet prepared from frozen mango fruits. During this study, physical properties such as density, cryoscopic temperature, osmotic pressure, overrun and melting time were analyzed. In order to assess the crystal structure and its changes, microscope images were taken of each sample after 1, 30 and 90 days of storage. The stabilizers showed no significant effect on the physical properties of the ice cream mixture; however, the sample with iota carrageenan stood out for having the highest overrun (58.7%) and the sample with kappa carrageenan took the longest to melt of all tested samples (almost 21 min). This study shows a significant effect of carrageenans in reducing the initial size of ice crystals as well as reducing recrystallization during storage. The stabilizing blend using ι-carrageenan provided the most effective cryoprotective properties, with an ice crystal diameter of 9 µm.

## 1. Introduction

Sorbets are a large group of frozen products with features that are attractive to consumers, such as the feeling of refreshment during consumption, lightness or a low energy value. Their quality is influenced by many factors: the type of raw materials used for production, the course and parameters of the freezing process, and the stability of conditions and storage time. Products such as ice cream and sorbets are burdened with the risk of ice crystallization in the form of large crystals and recrystallization, i.e., increasing the size of crystals during storage by combining smaller crystals into larger clusters, which results in a feeling of sandiness when consuming such a product. This can be prevented by using food additives from the group of stabilizers, called cryoprotectants [1,2,3,4,5].

Ice cream stabilizers such as guar gum, locust bean gum, xanthan and carrageenans are designed to help form a favorable ice cream structure and maintain it during storage. Carrageenan is a hydrocolloid obtained from seaweed. It has five fractions: kappa, iota, lambda, mi and ni, of which the first two fractions are the most popular in ice cream production. Carrageenan, after hydration, forms gels in some cases even after heating and cooling. In this way, free water is bound, which limits its migration in the product, placing a limitation on ice crystal growth while freezing and storing. Thanks to this, the obtained product is characterized by a smooth and fluffy consistency. Carrageenans are easily available stabilizers, and in addition, they show synergistic effects with other substances, which allows for the design of cryoprotective mixtures best suited to different types of ice cream and sorbets [6,7].

Many studies suggested that some aspects of stabilizer functionality with respect to recrystallization protection come from the structure, as measured by rheological properties, resulting from the freeze concentration of the polysaccharide in the unfrozen phase of the ice cream. The structure from the stabilizers will affect the rate at which water can diffuse to the surface of growing ice crystals [8]. One of the first stabilizers used in the formulation of ice creams was gelatine. Its effect has been explained as a mechanical interference with ice growth. Researchers [9] explained this behavior, suggesting that a firm gel would be more fragile and could be ruptured more easily by the ice front, while a more flexible gel would probably exert a stronger opposing force for ice front propagation. It has been reported as well that stabilizers that did not form a gel could still retard ice crystal growth [10]. It was determined [11,12] that some non-gelling systems showed lower recrystallization rates than the systems where a gel-like structure was observed. These results suggest that the steric blocking of the interface, or the inhibition of the solute transport to and from the ice interface, caused by the gelation of the polymer, is not the only mechanism of stabilizer action. The most efficient stabilizer will give sufficient microviscosity to the solution and steric hindrance to retard water diffusion to other crystals and, at the same time, it will need to have high flexibility properties that are not affected by heat shock.

Kappa and iota carrageenans are most commonly used to stabilize frozen products and desserts. These fractions differ in their molecular structure. The kappa fraction contains one sulfite group per disaccharide, and the iota fraction two, which is why they exhibit different stabilizing properties. Kappa carrageenan reacts with potassium ions, creating a brittle and hard gel that is stable while freezing and thawing. The iota fraction, on the other hand, reacts with calcium ions, which results in the formation of a soft, elastic gel. This fraction acts synergistically with locust been gum, also known as carob gum, but such a combination is susceptible to syneresis. The reaction with calcium ions causes ι-carrageenan to form a gel network resistant to phase separation, which is why this fraction is mainly used in the production of milk ice cream. Both the iota and kappa fractions are thermo-reversible, i.e., they can dissolve after heating and gel after cooling again [6,13].

What is also of great importance in technology is the λ (lambda) fraction, which dissolves in cold water but does not form a gel, unlike the κ (kappa) and ι (iota) fractions, which are soluble in water after heating to 60–75 °C. However, this fraction has thickening properties, which give a creamy effect in milk desserts. The greater thickening than the gelling ability of lambda carrageenan results from its low reactivity towards proteins. This fraction also shows solubility in cold milk, which translates into a wide range of applications in the dairy industry, but unfortunately it is not resistant to freezing and thawing. In addition, lambda carrageenan does not show synergistic properties with any other hydrocolloid [5,14].

It has already been discovered that there is no correlation between the viscosity and ice recrystallization inhibition (IRI) activity of different fractions of carrageenan [6]. It was also proven that stabilizing substances obtained by the acid and enzymatic hydrolysis of kappa and iota carrageenan could beneficially limit the recrystallization process in model sucrose solutions and ice creams. SEC (Size exclusion chromatography) and FTIR (Fourier-transform infrared spectroscopy) analyses confirmed that IRI activity in the recrystallization process is influenced by the structure of hydrolysates and the functional groups present, not only by the reduction in their molecular weight [15]. In this study, the influence of kappa iota and lambda fractions on the physical properties and crystal structure of stored mango sorbet was investigated to determine if the gelling fractions (iota and kappa) have a better impact on IRI activity than the thickening but nongelling lambda fraction. In the literature, we found studies on the lambda fraction only in model solutions [3] and not in a specific product; therefore, there seemed a need to investigate whether this fraction can be used as an effective stabilizer in different types of ice cream.

## 2. Results and Discussion

### 2.1. Physical Properties of Sorbet

#### 2.1.1. Osmotic Pressure and Cryoscopic Temperature

The key parameter that has a direct impact on the freezing process is cryoscopic temperature. The cryoscopic temperature value is a key parameter influencing the sensory quality of edible ice cream, as it directly affects the course of ice crystallization. When the value of the ice crystallization point is known, one can determine the optimal conditions for freezing and storing the product. A lower value of this parameter allows for obtaining smaller ice crystals in the product, which gives the ice cream a higher sensory quality [13,16,17]. It is lowered by dissolved substances, such as sucrose. Hydrocolloid addition usually does not affect the change in osmotic pressure and therefore does not significantly affect the reduction in cryoscopic temperature; however, different hydrocolloids may have different effects on the organization of water molecules in the crystal lattice and thus on the size and distribution of ice crystals in the product [3,15,17].

The samples stabilized with kappa and iota carrageenan were characterized by a lower osmotic pressure than the other variants (Table 1), at 1566.2 mOsm/kg and 1449.5 mOsm/kg, respectively. It was found that both fractions reduced recrystallization in model solutions, but neither exhibited thermal hysteresis activity [6]. That also suggested that their influence on cryoscopic temperatures was none or minimal.

The highest osmotic pressure in the conducted studies was measured in the sorbet with a λ variant addition. It amounted to 1651.8 mOsm/kg, with a cryoscopic temperature of −3.069 °C. It was also discovered that the lambda fraction as a popular densifier gives a higher viscosity to the model solutions even though it has no gelling properties. The influence of the λ fraction on cryoscopic temperature depression could suggest a better influence on ice recrystallization inhibition; however, some researchers claimed that there is no correlation between viscosity and the IRI activity of carrageenans [3].

#### 2.1.2. Density

The addition of stabilizing substances to the ice cream mass has a significant impact on both the finished product, such as sorbets, and the semi-finished product in the form of pasteurized and cooled ice cream mass [13,16]. The literature density of the ice cream mix can range from 1.0544 to 1.1232 g/cm^3^ and it is usually lower for the sorbet type of ice cream; however, an increase in the density of the ice cream mixture is an undesirable phenomenon because it makes it difficult to obtain the appropriate overrun of the product [18]. A one-way analysis of variance showed no significant statistical differences in the density of the analyzed sorbet samples (Table 2); however, the highest density value (1.177 g/cm^3^) was noted for the kappa carrageenan addition and the lowest, lower even than for the control sample (1.159 g/cm^3^, while for control sample, 1.168 g/cm^3^), for the addition of the λ fraction. This observation could suggest that stabilizer addition has a slight impact on product density [17,19,20].

#### 2.1.3. Overrun and Melting Time

Adsorbed particles of stabilizers could affect the viscosity of the final product. Some hydrocolloid additions may make it difficult to incorporate air into the structure while freezing, which means that the overrun of the product with the addition of stabilizing substances will be lower. The overrun of ice cream except for the addition of stabilizers depends on the basal recipe of ice cream. Theoretically, better air incorporation is possible in milky ice cream with a high fat content [21]. This research showed that overrun close to the milky ice cream level (50–60%) was noted for the sample with the addition of ι and κ fractions (Table 3). The capacity for air incorporation probably also depends on the basal fruit for sorbet preparation [13,20,22].

The overrun of stabilized ice cream may decrease during storage, which was confirmed by Palka and Wilczyńska [23]. They examined industrial sorbet, made from mango puree (13%) and passion fruit juice (12%), also containing LBG and guar gum, as well as emulsifiers in the form of mono- and diglycerides of fatty acids. At the beginning of this study, the overrun was at a level of 71.24%, and after six months of storage, it decreased to about 52%. This shows that the use of stabilizers allows for a high overrun value just after production. However, during storage, the crystal structure is not able to hold as much air, and as a result, the level of air incorporation decreases over time.

Extending the melting time is a particularly important parameter for the consumer due to the possibility of peaceful consumption on hot days or bringing ice cream home from the store [17,24]. Table 3 shows the time until the first drop of dissolved sorbet appears at an ambient temperature of 25 °C. The sorbet without stabilizers melted the fastest, and the time until the first drop appeared was close to 18 min. A similar result was obtained for the samples with ι and κ carrageenan addition—over 19 min. The longest time was obtained in the case of the sample with λ carrageenan used, which was statistically different from the sample without the addition of stabilizing substances (Table 3). According to the literature, there is a relationship between melting time and the overrun parameter, whereby the fluffier the ice cream, the slower it melts. This is because air bubbles act as insulators and limit heat transfer to the ice cream [25]. However, it was also discovered that for sorbet, an inverse relationship can be observed, which is related to the lack of fat globules and the presence of monosaccharides, which cause the sorbet to melt faster [13].

Petkova et al. [22] decided to investigate this attribute using peach sorbet with the addition of *Zizyphus jujuba* powder (this fruit is well-known on the Chinese market). The ice cream melting test was conducted at an ambient temperature of 22 °C for 120 min, and the mass of melted ice cream was weighed every 5 min. The first signs of melting appeared between the 10th and 15th min, and the ice cream was completely dissolved around the 19th min. In line with that result, carrageenans seem to be a better idea for stabilizing a sorbet type of ice cream.

### 2.2. Crystal Structural Analysis

Controlling the formation and growth of ice crystals is crucial to achieving the desired consistency of ice cream. Ice crystal growth in the product leads to an increase in the hardness of the ice cream, making the product less attractive to the consumer [11,26].

**Figure 1:**
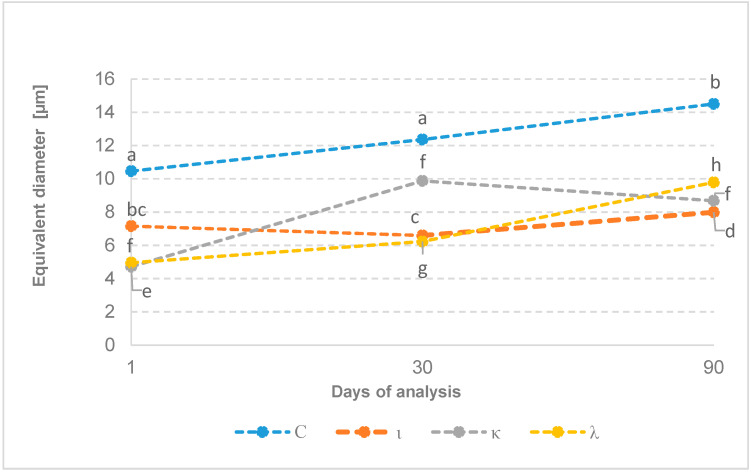
Ice crystal size distribution over time: C—control sample, ι—iota carrageenan, κ—kappa carrageenan, λ—lambda carrageenan; letters a–h mean values denoted by different superscripts differ statistically at α = 0.05.

The analysis of the ice crystal size distribution in the sorbets shows that, in the case of the control sample without added stabilizers, the increase in diameters is noticeable and statistically significant, from 10.5 µm after 1 day of storage to 14.5 µm after 90 days of storage (Figure 1, Table 4).

Variant ι showed the highest value of the equivalent diameter at 1 day among the stabilized samples. It was 7.2 µm; however, it was not statistically significantly different from the same sample tested after 30 and 90 days of storage at a temperature of −18 °C, which may indicate the effective inhibition of the recrystallization process by the iota fraction. According to Hale et al. [2], iota fraction carrageenan has good recrystallization-inhibiting properties, but not as good as κ-carrageenan. The presence of calcium cations is beneficial in improving stabilizing properties. During tests of the effectiveness of carrageenans and their hybrid blends on the effectiveness of limiting recrystallization in model solutions of sucrose with the addition of calcium or potassium ions, it was shown that in the comparison of crystal size over 4 and 72 h of storage at −12 °C, the increase in the sample with the addition of calcium cations was statistically insignificant. However, in the case of the variant with the addition of potassium ions, the difference in crystal growth turned out to be statistically significant [2,27].

The sorbet stabilized with kappa carrageenan after 1 day had a crystal diameter of 4.7 µm and achieved significant crystal growth after 30 days (Figure 1, Table 4). The average equivalent crystal diameter was then 9.9 µm, which was statistically similar to ice crystals in the control sample after 1 day of storage; however, further growth was not noted even after 90 days of storage. Kamińska-Dwórznicka et al. [1] studied the effect of kappa carrageenan and its hydrolysates on limiting the growth of ice crystals in strawberry sorbet. The stabilizing mixture used consisted of 0.125% gelatin, 0.125% xanthan gum, and 0.01% κ-carrageenan. It was shown that just after 1 month of storage at −18 °C, an equivalent crystal diameter in the examined strawberry sorbet was at a level of 25 µm. This effect could be caused by the mix of stabilizers or it could suggest that different types of fruits in sorbet could also influence the progress of the recrystallization process.

The ice crystals tested with the addition of the λ fraction after 1 day were statistically similar to the crystals in the sample with the κ fraction; their mean equivalent diameter was 5 µm. Also, for the sample with λ addition, the ice crystal growth after 1 month of storage was not significant (Figure 1); however, after 90 days of storage, the equivalent diameter was 10 µm. None of the analyzed samples exceeded the ice crystal detection threshold (between 25 and 50 µm, according to [8,11]), even after 90 days of storage (Figure 1, Table 4).

**Figure 2:**
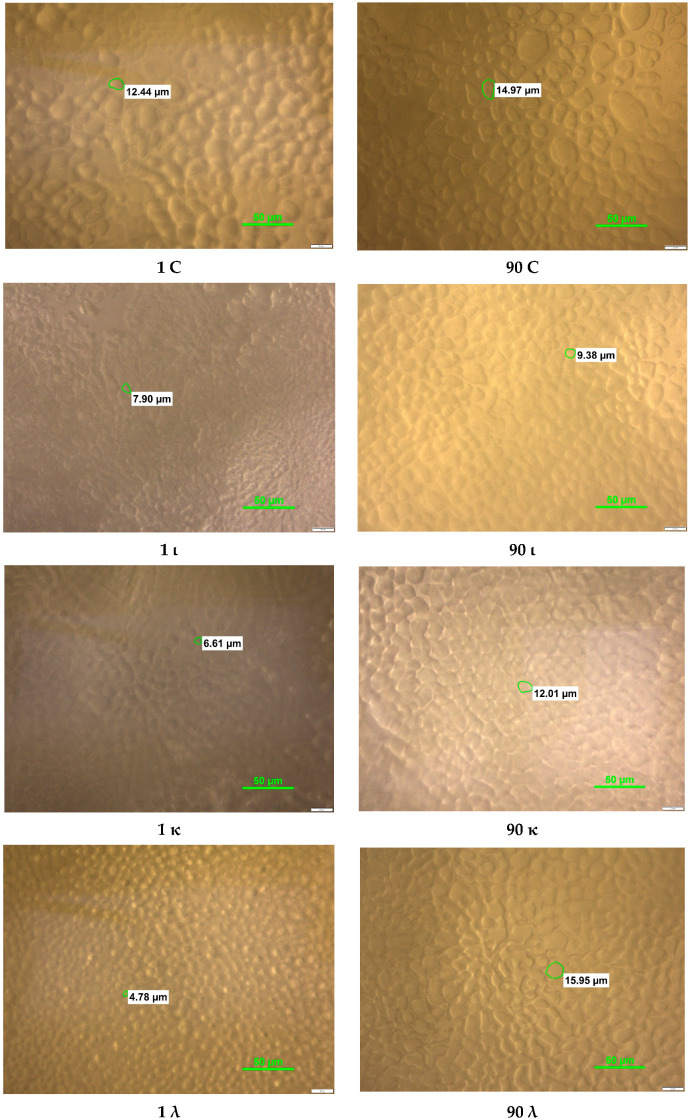
Images for all variants: C—control sample, ι—iota carrageenan addition, κ—kappa carrageenan addition, and λ—lambda carrageenan addition; after 1 and 90 days of storage at −18 °C.

After the first day of storage, the stabilized sorbets had more regular crystal shapes than the control sorbet (Figure 2).

After 90 days of storage, the shape of the ice crystals in all samples tested became more irregular, and the space between the crystals increased, which was most noticeable for the λ variant. The occurrence of elongated shapes of ice crystals during storage proves the phenomenon of recrystallization, which consists of combining small crystals into larger clusters, which is mainly caused by the phenomenon of coalescence. The influence of stabilizers on crystal morphology is also confirmed by Kamińska-Dwórznicka et al. [13]. In the study of strawberry sorbet, they obtained smaller crystals with more regular shapes, thanks to the use of freezing in a cryostat and 0.1% stabilizers in the form of kappa and iota carrageenans and their mixture in a 1:1 ratio, where the most regular shape with the smallest size was obtained using iota carrageenan, which confirms the morphology of the tested crystals presented in Figure 2. Kappa carrageenan used alone resulted in the formation of regular shapes of comparable size. In the case of the sample stabilized with iota fraction carrageenan, the obtained crystals were characterized by an irregular shape, and the spaces between them were large. However, each variant of stabilizers caused a significant reduction in ice crystallization compared to the control sample, in which the ice crystals were very large and irregular in shape [13,28].

## 3. Conclusions

The results obtained show that the best sorbet properties were obtained in the stabilizing mixture with the iota fraction of carrageenan used.

Iota addition gives the highest overrun of sorbet, while the lowest overrun was obtained in the control sample. The longest melting time was obtained in the variant with added carrageenan λ, 21 min. The shortest melting time was measured in the control sample and was 17 min.

All carrageenans showed a significant effect in limiting ice crystallization and recrystallization in sorbets. The lowest average crystal size after 90 days of storage was shown by the iota fraction carrageenan (around 8 µm) followed by kappa (around 9 µm). On the other hand, the λ fraction carrageenan was characterized by the slight IRI activity, which may exclude this variant from the group of effective stabilizers. During storage, the variant using iota carrageenan was characterized by the greatest resistance to changes in crystal morphology in the form of an increase in size and loss of shape regularity. Moreover, it can be expected that composing new stabilizing mixtures based on tested carrageenans and other additives can result in obtaining a stabilizer combining the best possible properties. The variant with the largest crystals obtained—the control sample after 90 days of storage—was characterized by an average equivalent diameter of 14.5 μm, which gives a much lower result than the declared detection threshold (25–50 µm). This indicates the natural properties of the original recipe used limit recrystallization, which can be used in the production of sorbet without any stabilizing substances. That could also suggest lower costs of sorbet production.

## 4. Materials and Methods

### 4.1. Materials and Sorbet Preparation

The total addition of stabilizers in the sorbets was at a level of 0.3%, including 0.1% guar gum (Agnex, Białystok, Poland), 0.1% LBG (Agnex, Białystok, Poland) and 0.1% as a variable element carrageenan of fractions κ, ι and λ (all fractions Sigma-Aldrich/Merck, Darmstadt, Germany, distribution Warsaw, Poland). In addition, a control sample (C) was prepared without the use of stabilizers, and the missing mass was supplemented with water. The recipe for the sorbet was based on frozen mango fruits—Hortex, Siemiatycze Poland (66%) with the addition of sucrose (13.8%); mango syrup—Monin, US, Clearwater, Florida, distribution Warsaw, Poland (5.5%); and water (13.9%—for stabilized samples; 14.2%—for non-stabilized samples).

Frozen mango fruit in pieces (Hortex, Siemiatycze, Poland) was thawed, and the remaining ingredients were added and then crushed with a Bosch hand blender until a smooth mass was obtained. The obtained mixture was pasteurized in a Thermomix device at 72 °C for 15 s. The prepared system was cooled to an ambient temperature, after which samples were taken for analysis. The remaining part of the mixture was transferred to a G3 Ferrari G20035 classic freezer, where the freezing process was carried out for 50 min, during which the temperature changes were controlled using two thermocouples cooperating with an MPI-LAB device. Readings were recorded every 2 min. Sorbets in each variant were prepared in triplicate.

### 4.2. Physical Properties’ Analysis

#### 4.2.1. Cryoscopic Temperature and Osmotic Pressure

Cryoscopic temperature and osmotic pressure were determined using a Marcel OSM 3000 (Marcel S.A., Zielonka, Poland) osmometer designed to measure both parameters. Approximately 100 µL of the mixture was transferred to Eppendorf tubes, and the measurement lasted until the device stabilized. The measurement accuracy was 0.002 °C, the determination was performed in triplicate and, for each sample, both parameters were taken two times [29].

#### 4.2.2. Density

The density of the sorbet mixture before freezing was measured using a 25 cm^3^ pycnometer and a laboratory scale. The determination was performed in triplicate [17].

#### 4.2.3. Overrun and Melting Time

In order to calculate the overrun of the sorbet, the prepared mixture before freezing was transferred to a 25 cm^3^ cylinder, and the accurate mass was read. The procedure was repeated with the sorbet after freezing in the freezer, and the overrun was then calculated as a percentage, as already described in a previous study [17,30]. The determination was performed in triplicate.

After freezing, the sorbet was transferred to a metal cylinder at about 20 g, and then the samples prepared in this way were stored for 24 h at a temperature of −18 °C. After the appropriate time had elapsed, the cylinders were taken out to room temperature, and the time until the first drop of melted sorbet flowed down from the cylinder placed on a glass funnel was measured. The measurement was performed in duplicate based on a previous study [16,17,31].

### 4.3. Crystal Structural Analysis

The obtained sorbets were stored for 1, 30 and 90 days. Every time, each sample was subjected to computer image analysis using an Olympus BX53 microscope (Tokyo, Japan, distribution Warsaw, Poland) equipped with a Linkam LTS420 cooling system (Redhill, UK) and an Olympus SC50 camera (Tokyo, Japan, distribution Warsaw, Poland). After taking images (at least 12 for each sample, after each storage time), 300 ice crystals in each sample were outlined using the NIS Element D program, and then the area, equivalent diameter and standard deviation of examined crystals were calculated based on a previous method [1].

### 4.4. Statistics

All calculations according to the presented formulas were carried out using Microsoft Excel 2021. Also, using this program, graphs regarding the distribution of ice crystal sizes in the analyzed samples were produced [1].

Statistical analysis was performed using the Statistica 13.3 program with the assumed significance level of α = 0.05. The physical parameters of the sorbet—density, osmotic pressure, cryoscopic temperature, overrun and melting time—were subjected to one-way analysis of variance with Tukey’s test. On the other hand, the equivalent diameters of the measured ice crystals were subjected to two-way analysis of variance with Tukey’s test, where the qualitative factors were the type of stabilizer used and the storage time.

## Figures and Tables

**Table 1:** Average osmotic pressure and average cryoscopic temperature of sorbet mix.

Variant	Osmotic Pressure [mOSM/kg]	Cryoscopic Temperature [°C]
C	1617.2 ± 106.5 ^a^	−2.991 ± 0.188 ^a^
ι	1449.5 ± 159.6 ^a^	−2.693 ± 0.297 ^a^
κ	1566.3 ± 93.3 ^a^	−2.910 ± 0.173 ^a^
λ	1651.8 ± 56.3 ^a^	−3.069 ± 0.104 ^a^

C—control sample, ι—iota carrageenan, κ—kappa carrageenan, λ—lambda carrageenan; ($\bar{x}$ ± sd); the same superscript “^a^” means that the mean values are not statistically different at α = 0.05 statistically at α = 0.05.

**Table 2:** The average density of a sorbet mix.

Variant	Density [g/cm^3^]
C	1.168 ± 0.013 ^a^
ι	1.162 ± 0.020 ^a^
κ	1.177 ± 0.024 ^a^
λ	1.159 ± 0.019 ^a^

C—control sample, ι—iota carrageenan, κ—kappa carrageenan, λ—lambda carrageenan; ($\bar{x}\;$± sd) the same superscript “^a^” means that the mean values are not statistically different at α = 0.05; α = 0.05.

**Table 3:** The average fluffiness and average melting time of a sorbet mix.

Variant	Overrun [%]	Melting Time [min]
C	30.59 ± 0.32 ^a^	17.45 ± 0.75 ^a^
ι	58.72 ± 2.13 ^b^	19.45 ± 0.25 ^ab^
κ	49.16 ± 8.14 ^ab^	19.43 ± 0.32 ^ab^
λ	35.15 ± 2.04 ^ab^	21.14 ± 0.18 ^b^

C—control sample, ι—iota carrageenan, κ—kappa carrageenan, λ—lambda carrageenan; ($\bar{x}$ ± sd); ^a–ab^ mean values denoted by different superscripts differ statistically at α = 0.05.

**Table 4:** Equivalent diameter for each variant of sorbet and each variant of storage.

Variant	Equivalent Diameter [µm]
1 C	10.46 ± 1.67 ^a^
1 ι	7.16 ± 1.15 ^bc^
1 κ	4.72 ± 1.06 ^e^
1 λ	4.96 ± 2.00 ^f^
30 C	12.36 ± 3.12 ^a^
30 ι	6.59 ± 1.12 ^c^
30 κ	9.87 ± 2.14 ^f^
30 λ	6.23 ± 2.07 ^g^
90 C	14.50 ± 3.02 ^b^
90 ι	7.99 ± 0.83 ^d^
90 κ	8.71 ± 2.14 ^f^
90 λ	9.79 ± 1.04 ^h^

C—control sample, ι—iota carrageenan, κ—kappa carrageenan, λ—lambda carrageenan; ($\bar{x}$ ± sd); ^a–h^ mean values denoted by different superscripts differ statistically at α = 0.05.

## Data Availability

The data generated or analyzed during this study are available from the corresponding author on reasonable request.
